# EXPLORING THE ASSOCIATION BETWEEN GOOGLE RATINGS AND NURSING HOME QUALITY

**DOI:** 10.1093/geroni/igae098.3165

**Published:** 2024-12-31

**Authors:** Neeraj Dayama, Shivani Gupta, Rohit Pradhan

**Affiliations:** Texas Tech University Health Sciences Center, Lubbock, Texas, United States; University of Houston - Clear Lake, Houston, Texas, United States; Texas State University, San Marcos, Texas, United States

## Abstract

Online review platforms such as Google ratings have become a resource for consumers seeking nursing home (NH) care for their loved ones. As these ratings reflect subjective experiences, it is important to explore how they may correlate with the more rigorous and systematic measures of NH quality. The primary purpose of this study was to explore the association of Google ratings with NH deficiencies. This study is structured as a cross-sectional analysis (2018) utilizing the following datasets: Brown University’s LTC Focus, Ohio nursing home Google rating data, and Area Health Resource File. The dependent variable was deficiencies which reflect a facility’s failure to meet specified resident care standards, known as federal participation requirements (FPRs). Examples of deficiencies include a facility’s failure to adhere to infection control protocols or improper medication management. The independent variable was consumer reported Google ratings for Ohio NHs. We used negative binomial linear regression, adjusting for NH organizational (size, occupancy, ownership, chain affiliation, payer mix) and market factors at the county level (per capita income, urban/rural location) to assess the relationship. Mean Google rating was 3.55 out of 5 and mean total deficiency count was 11.98. We found that Google ratings were negatively associated with deficiencies (Incidence Rate Ratio=0.93, p< 0.05) (as the number of deficiencies increased, Google ratings declined). From a policy perspective, our findings highlight the need for regulatory frameworks to incorporate consumer feedback mechanisms in evaluating and improving nursing home care quality.

